# Supplementary material for: A pilot study of neoadjuvant combination of anti-PD-1 camrelizumab and VEGFR2 inhibitor apatinib for locally advanced resectable oral squamous cell carcinoma
Source: Nat Commun. 2022 Sep 14;13:5378. doi: 10.1038/s41467-022-33080-8 (PMC9472189; doi:10.1038/s41467-022-33080-8)
Supplement: Supplementary file 1 — Supplementary Information [file 41467_2022_33080_MOESM1_ESM.pdf]

Index

Supplementary Table 1. All neoadjuvant therapy-related adverse events and surgical-related adverse events 2
Supplementary Table 2. Radiotherapy-related adverse events in 18 participants underwent radiotherapy 4
Supplementary Table 4. Information of 20 Patients for Efficacy Analysis 6
Supplementary Table 5. Pathological Response in Metastatic Lymph Nodes 8
Supplementary Table 6. The definition of RVT% and MPR. 9
Supplementary Table 7. The definition of immune-related pathologic tumour bed characteristics in oral squamous cell carcinoma and corresponding figure numbers. 10
Supplementary Figure 1. Representative immune-related pathologic tumour bed HE pictures (Case No.6). 11
Supplementary Figure 2. Fluorescence intensity quantification and ratios of tumour-infiltrating lymphocytes (TILs) in tumour tissues before- and after- neoadjuvant therapy. 12
Supplementary Figure 3. Comparison of changes (Δ) of TILs over the neoadjuvant therapy between MPR and non-MPR groups 13
Supplementary Figure 4. Comparison of TILs over the neoadjuvant therapy and between MPR and non-MPR groups. 14
Supplementary Figure 5. Flow of slides preparation. 15
Supplementary Figure 6. Paraffin embedded slides. 16
Supplementary Figure 7. Representative immune-related pathologic tumour bed HE pictures (Case No.7). 17
Supplementary Figure 8. Representative immune-related pathologic tumour bed HE pictures (Case No.1). 18
Supplementary Figure 9. Representative immune-related pathologic tumour bed HE pictures (Case No.8). 19
Supplementary Note 1. Study protocol 20

Supplementary Table 1. All neoadjuvant therapy-related adverse events and surgical-related adverse events

Case No.	Skin (rash, dryness, dermatitis)	Pain (lymph node and oral)	Colitis/ Diarrhea	Fatigue	Proteinuria	Hypertension	Hyperbilirubinemia	Thrombocytopenia	Leukopenia	Increased AST level	Reactive capillary hemangiomas	Surgical-Related Adverse Events (Clavien-Dindo grade)
1						**G2**				**G1**		
2										**G1**		
3							**G1**				**G1**	Subcutaneous exudate (G1)
5						**G1**						
6		**G1**						**G1**				Post-tracheostomy bleeding (G1)
7												
8				**G1**	**G2**						**G1**	
9			**G1**	**G1**	**G1**				**G2**			
10		**G1**					**G1**	**G1**				
11		**G1**					**G1**				**G1**	
12	**G2**						**G2**	**G1**				
13							**G1**					
14		**G2**				**G2**	**G1**			**G1**		
15					**G2**	**G2**	**G1**	**G1**	**G1**			
16					**G1**			**G2**				
17				**G1**	**G1**				**G1**			
18			**G1**		**G2**		**G1**	**G1**				

Case No.	Skin (rash, dryness, dermatitis)	Pain (lymph node and oral)	Colitis/ Diarrhea	Fatigue	Proteinuria	Hypertension	Hyperbilirubinemia	Thrombocytopenia	Leukopenia	Increased AST level	Reactive capillary hemangiomas	Surgical-Related Adverse Events (Clavien-Dindo grade)
19								**G1**				Post-flap-reconstruction pharyngeal fistula (G1)
20												
21								**G1**				Wound infection (G1)

All neoadjuvant therapy-related adverse events were graded according to Common Terminology Criteria for Adverse Events Version 5.0.

Abbreviations: G1: Grade 1. G2: Grade 2. AST, aspartate aminotransferase.

Supplementary Table 2. Radiotherapy-related adverse events in 18 participants underwent radiotherapy

Radiotherapy-Related Adverse Events*	*N* (%)		
	Grade 1-2	Grade 3	Grade 4
Radiodermatitis	12 (66.7)	2 (11.1)	
Radiation-induced oral mucositis	16 (88.9)		
Leukopenia	4 (22.2)		
Hemoglobin decline	8 (44.4)		
Thrombocytopenia	5 (27.8)		
Increased AST level	2 (11.11)		
Hyperbilirubinemia	3 (16.7)		

*Radiotherapy-related adverse events were graded according to Common Terminology Criteria for Adverse Events Version 5.0.

Abbreviations: AST, aspartate aminotransferase.

Supplementary Table 3. Pathological response

Patient No.	Baseline Biopsy Sample					Surgical Resected Sample	
	TPS	CPS	CPS ≥ 1	CPS > 10	CPS ≥ 20	%RVT	MPR[a]
1	0	0				7.36	+
2	0	0				29.41	
3	0	1	+			27.17	
5	5	8	+			62.13	
6	20	25	+	+	+	9.37	+
7	3	3	+			8.76	+
8	30	40	+	+	+	0.23	+
9	10	12	+	+		9.76	+
10	3	5	+			27.51	
11	30	35	+	+	+	1.94	+
12	0	2	+			40.52	
13	0	0				23.30	
14	3	5	+			41.52	
15	3	5	+			33.00	
16	2	10	+			36.79	
17	0	0				49.12	
18	5	7	+			1.06	+
19	1	1	+			39.51	
20	3	5	+			34.91	
21	NA	90	+	+	+	0.00	+

a: MPR: no more than 10% RVT.

Abbreviations: TPS, Tumour Proportion Score. CPS, Combined Positive Score. RVT, Residual Viable Tumour. MPR, Major Pathological Response.

Supplementary Table 4. Information of 20 Patients for Efficacy Analysis

Patient No.	Disease Stage[a]						Radiographic Response[c]			Pathological Response	
	Baseline cT stage	Baseline cN stage	Baseline cTNM	Baseline c stage	pTNM (post-neoadjuvant therapy)[b]	p stage (post-neoadjuvant therapy)[b]	Δ Primary Lesion (mm)	Δ Sum[d] (mm)	RECIST 1.1	RVT (%)	MPR[e]
1	3	0	T3N0M0	III	T2N1M0	III	-15.1	-15.1	**PR**	7.36	**+**
2	3	0	T3N0M0	III	T3N0M0	III	-2.1	-2.1	**SD**	29.41	
3	3	2C	T3N2M0	IVa	T3N3M0	IVb	-4.2	-4.8	**SD**	27.17	
5	3	0	T3N0M0	III	T3N0M0	III	0	0	**SD**	62.13	
6	3	2b	T3N2M0	IVa	T3N2M0	IVa	-5.2	-6	**SD**	9.37	**+**
7	3	1	T3N1M0	III	T2N1M0	III	-8.4	-11	**SD**	8.76	**+**
8	3	0	T3N0M0	III	T2N0M0	II	-6.5	-6.5	**SD**	0.23	**+**
9	3	0	T3N0M0	III	T3N0M0	III	14.7	14.7	**PD**	9.76	**+**
10	3	0	T3N0M0	III	T3N0M0	III	-2.1	-2.1	**SD**	27.51	
11	3	0	T3N0M0	III	T2N0M0	II	4.2	-12.2	**PR**	1.94	**+**
12	3	0	T3N0M0	III	T3N0M0	III	1.4	4.2	**PD**	40.52	
13	3	0	T3N0M0	III	T3N0M0	III	-12.2	1.4	**SD**	23.30	

Patient No.	Disease Stage[a]						Radiographic Response[c]			Pathological Response	
	Baseline cT stage	Baseline cN stage	Baseline cTNM	Baseline c stage	pTNM (post-neoadjuvant therapy)[b]	p stage (post-neoadjuvant therapy)[b]	Δ Primary Lesion (mm)	Δ Sum[d] (mm)	RECIST 1.1	RVT (%)	MPR[e]
14	3	0	T3N0M0	III	T3N2M0	IVa	14.3	14.3	**PD**	41.52	
15	3	0	T3N0M0	III	T3N0M0	III	NA	NA	**NA**	33.00	
16	3	1	T3N1M0	III	T3N3M0	IVb	7.5	9.4	**PD**	36.79	
17	3	0	T3N0M0	III	T3N2M0	IVa	11	11	**PD**	49.12	
18	4	0	T4N0M0	IVa	T3N0M0	III	-11.3	-15.2	**SD**	1.06	**+**
19	3	0	T3N0M0	III	T3N1M0	III	-10.2	-10.2	**SD**	39.51	
20	4	0	T4N0M0	IVa	T4N1M0	IVa	3.9	14.6	**PD**	34.91	
21	3	1	T3N1M0	III	T1N1M0	III	-14.3	-19.5	**PR**	0.00	**+ (pCR)**

[a] American Joint Committee on Cancer (AJCC), 8th Edition staging. [b] According to the AJCC principle, “pTNM” and “p stage” should be judged in primary tumours. Therefore, “pTNM” and “p stage” in this study, which were determined by samples underwent neoadjuvant therapy, need further discussion. [c] According to RECIST 1.1. [d] Diameter sum of target (primary) lesion and non-target lesion (cervical metastatic lymph node). [e] MPR: no more than 10% RVT.

Abbreviations: RECIST, Response Evaluation Criteria in Solid Tumours. PR, Partial Response. SD, Stable Disease. PD, Progressive Disease. NA, Not Available. RVT, Residual Viable Tumour. MPR, Major Pathological Response. pCR, Pathological Complete Response.

Supplementary Table 5. Pathological Response in Metastatic Lymph Nodes

Patient No.	pN stage	Response in lymph nodes	MPR [a] in primary tumour
1	1	Not significant	+
3	3b	Necrosis, Multinucleated giant cells, calcification	
6	2b	Necrosis, Multinucleated giant cells, Calcification	+
7	1	Necrosis, Multinucleated giant cells	
14	2c	Not Significant	
16	3b	Necrosis, Multinucleated giant cells	
17	2a	Necrosis	
19	1	Not Significant	
20	1	Necrosis, Multinucleated giant cells	
21	1	Necrosis, Proliferative fibrosis	+ (pCR)

[a] MPR: no more than 10% residual viable tumour.

Abbreviations: MPR, Major Pathological Response. pCR: Pathological Complete Response.

Supplementary Table 6. The definition of RVT% and MPR.

	Definition
"a"	residual viable tumour area
"b"	Necrosis area
"c"	regression bed area
Tumour bed	"a" + "b" + "c"
RVT%	"a" in all slides / ("a" + "b" + "c") in all slides
MPR	≤10% RVT

Abbreviations: RVT, Residual Viable Tumour. MPR, Major Pathologic Response.

Supplementary Table 7. The definition of immune-related pathologic tumour bed characteristics in oral squamous cell carcinoma and corresponding figure numbers.

Immune-related Pathologic Tumour Bed Characteristics in Oral Squamous Cell Carcinoma	Corresponding HE figures from this trial
Necrosis	Supplementary Figure 7
Multinucleated giant cells infiltration	Supplementary Figure 7
Proliferative fibrosis	Supplementary Figure 7
Dystrophic calcification	Supplementary Figure 8
Tumour infiltrating lymphocytes	Supplementary Figure 8
Neovascularization	Supplementary Figure 8
Foamy macrophages	Supplementary Figure 9
Dense plasma cells	Supplementary Figure 9
Tertiary lymphoid structure	Supplementary Figure 1

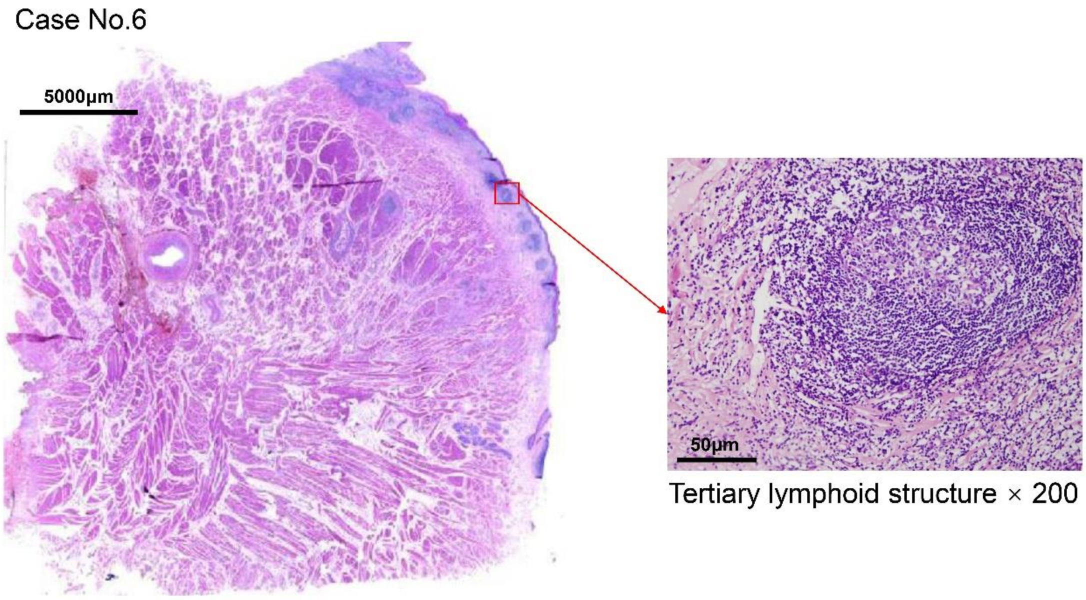

Supplementary Figure 1. Representative immune-related pathologic tumour bed HE pictures (Case No.6).

A representative H&E staining image of case No.6 shows: tertiary lymphoid structure. H&E, haematoxylin and eosin-stained.

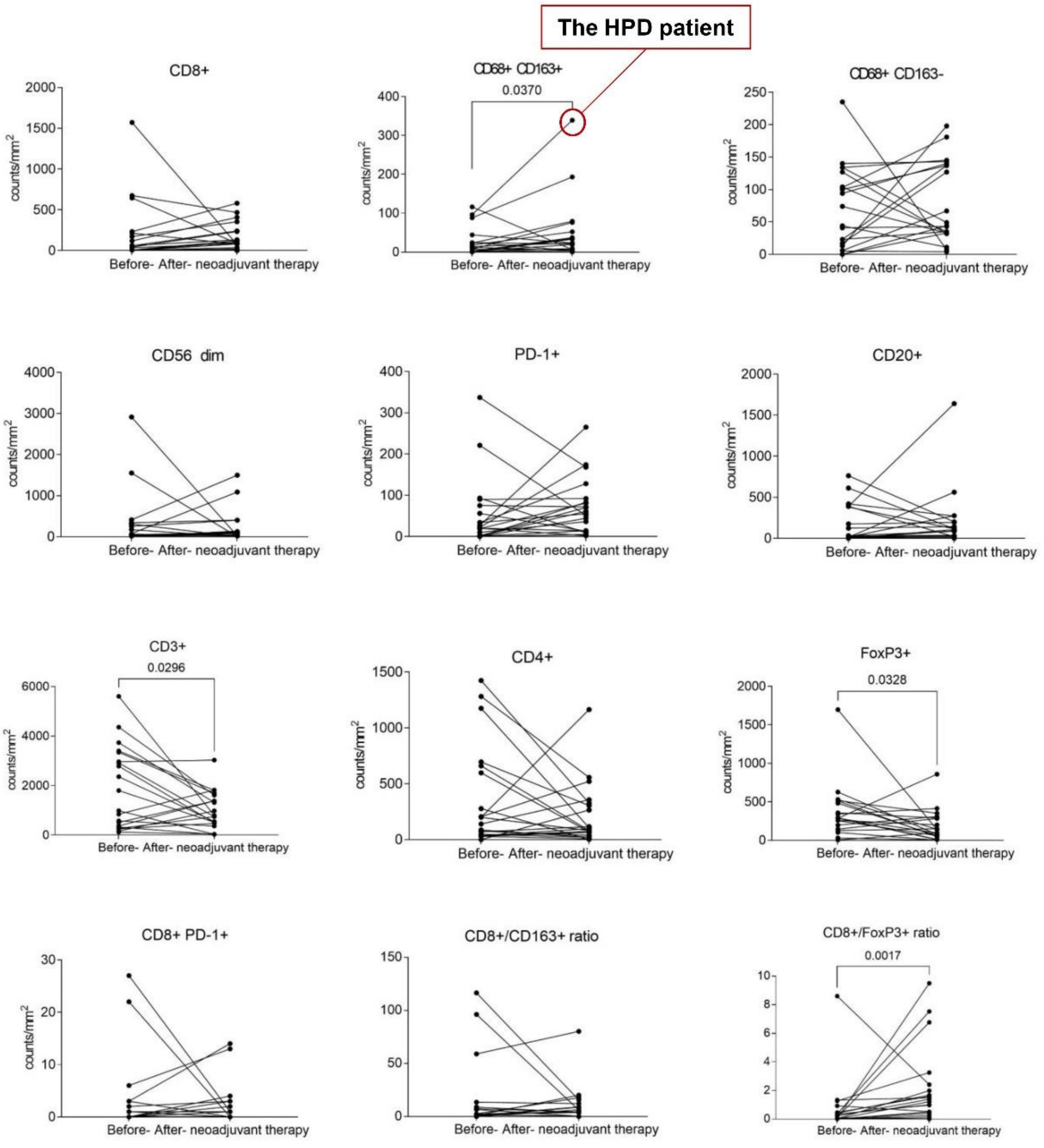

Supplementary Figure 2. Fluorescence intensity quantification and ratios of tumour-infiltrating lymphocytes (TILs) in tumour tissues before- and after-neoadjuvant therapy

A two-sided Wilcoxon signed-rank test was used and *p* values for significant results were presented in the figure (*n* = 20 pairs of tumours from patients completed neoadjuvant therapy and surgery). Source data are provided as a Source Data file. Abbreviations: HPD, hyperprogressive disease.

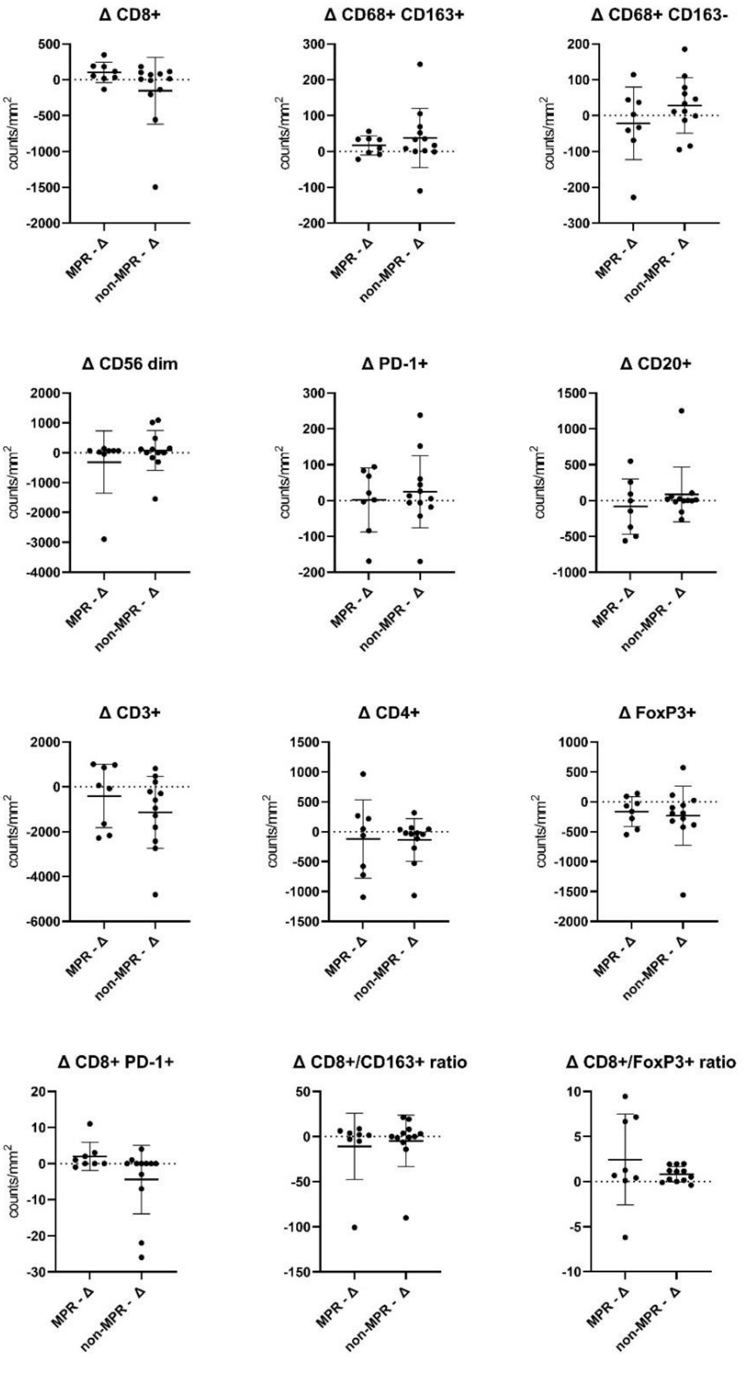

Supplementary Figure 3. Comparison of changes (Δ) of TILs over the neoadjuvant therapy between MPR and non-MPR groups

A two-sided Mann Whitney test was used and no significant result was found (MPR group [n = 8], non-MPR group [*n* = 12]). Bars represented mean with SD. Source data are provided as a Source Data file.

Abbreviations: MPR, major pathologic response.

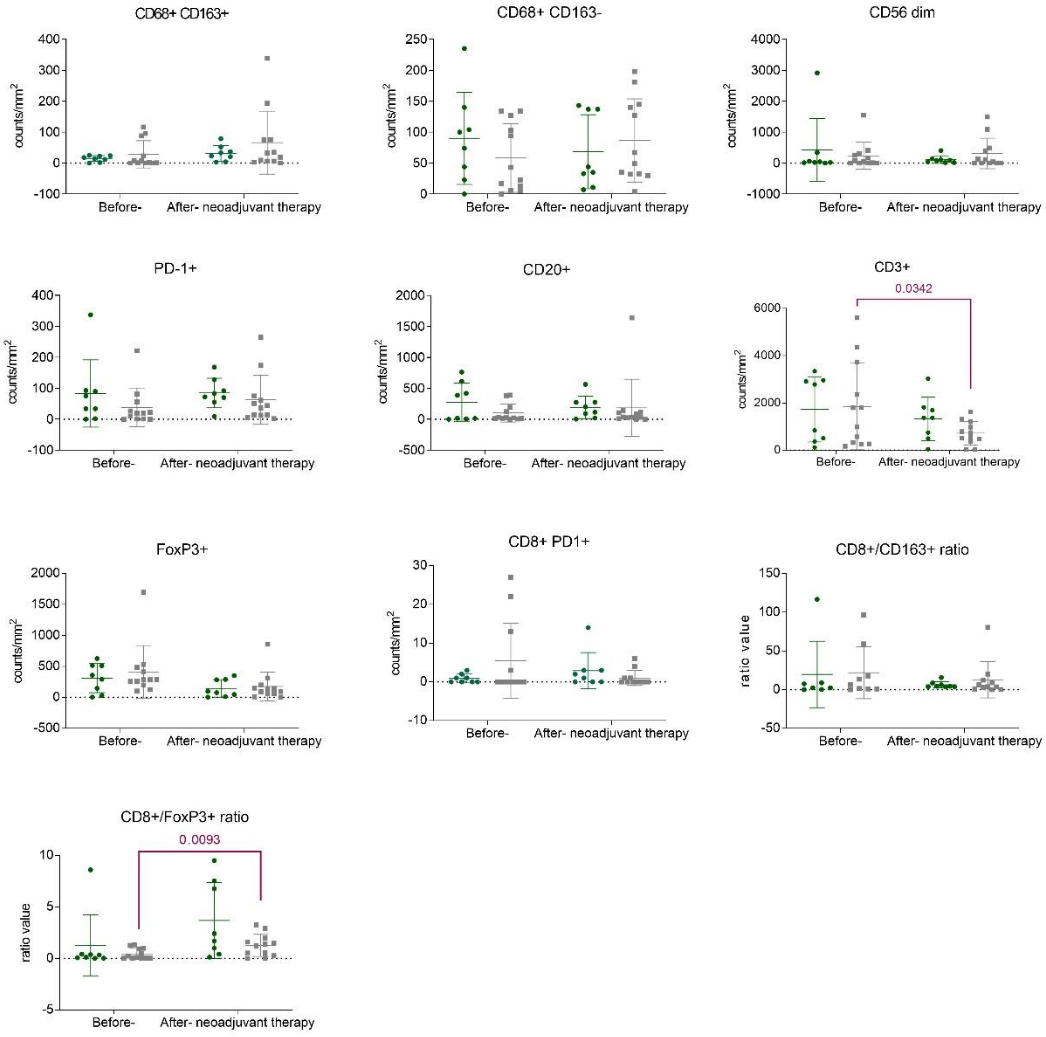

Supplementary Figure 4. Comparison of TILs over the neoadjuvant therapy and between MPR and non-MPR groups.

The significance for differences between before- and after- neoadjuvant therapy was tested using a two-sided Wilcoxon signed-rank test; for differences between MPR and non-MPR group, the significance was tested using a two-sided Mann Whitney test. *p* values for significant results were presented in the figure (green dots for the MPR group [n = 8], grey dots for the non-MPR group [n = 12]). Bars represented mean with SD. Source data are provided as a Source Data file.

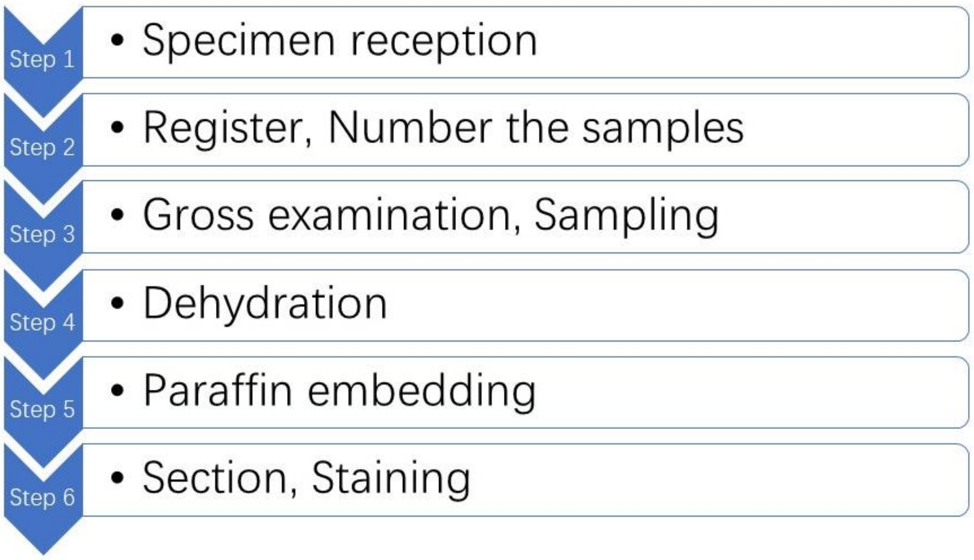

Supplementary Figure 5. Flow of slides preparation.

Schematic diagram of haematoxylin and eosin-stained slides preparation steps for tumor resection tissues from patients receiving neoadjuvant therapy.

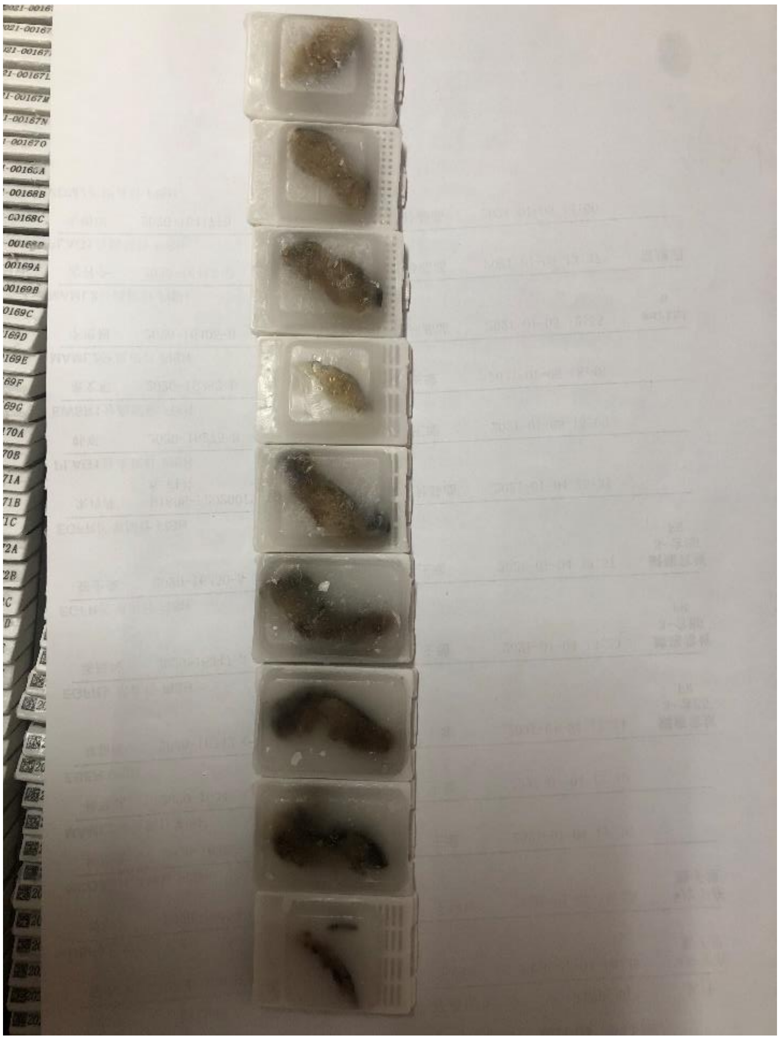

Supplementary Figure 6. Paraffin embedded slides.

Schematic diagram of formalin-fixed paraffin-embedded tissue blocks of tumor resection tissues from patients receiving neoadjuvant therapy.

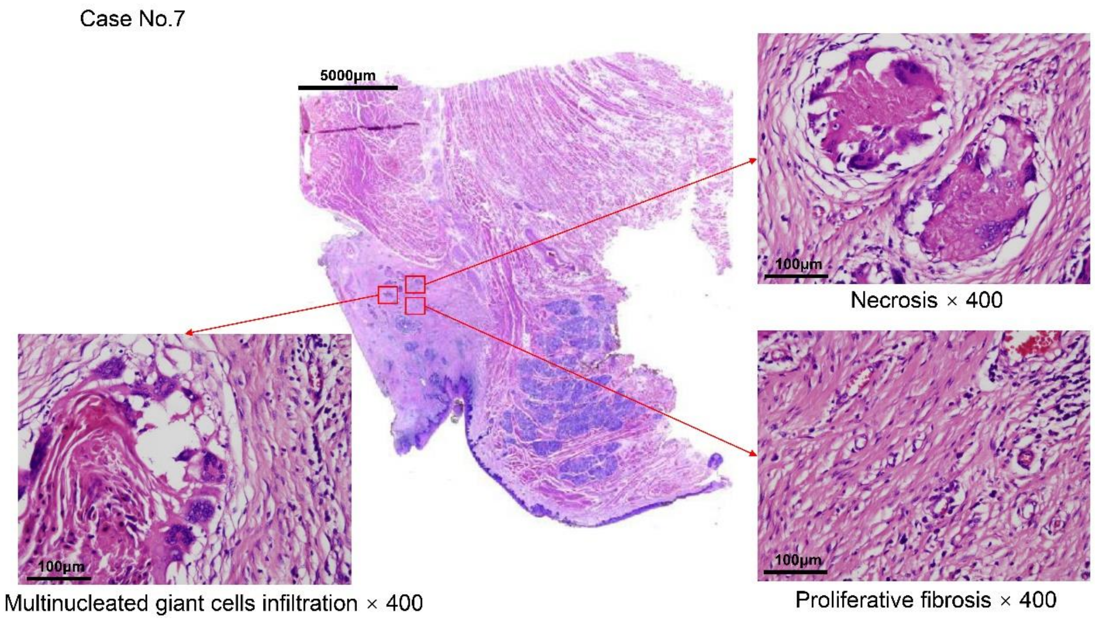

Supplementary Figure 7. Representative immune-related pathologic tumour bed HE pictures (Case No.7).

A representative H&E staining image of case No.7 shows: multinucleated giant cells infiltration, necrosis, and proliferative fibrosis. H&E, haematoxylin and eosin-stained.

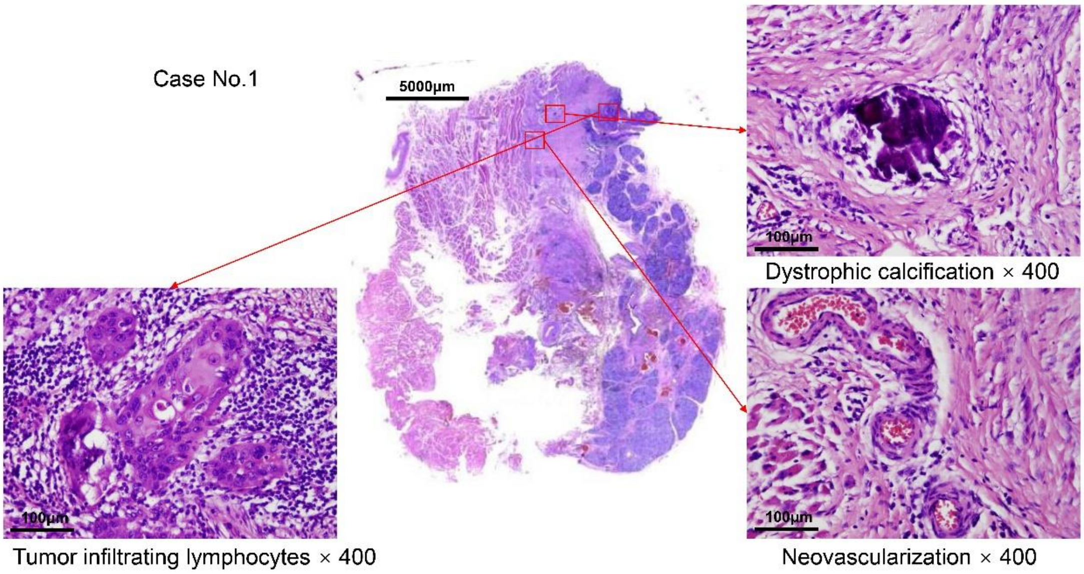

Supplementary Figure 8. Representative immune-related pathologic tumour bed HE pictures (Case No.1).

A representative H&E staining image of case No.1 shows: tumour infiltration lymphocytes, dystrophic calcification, and neovascularization. H&E, haematoxylin and eosin-stained.

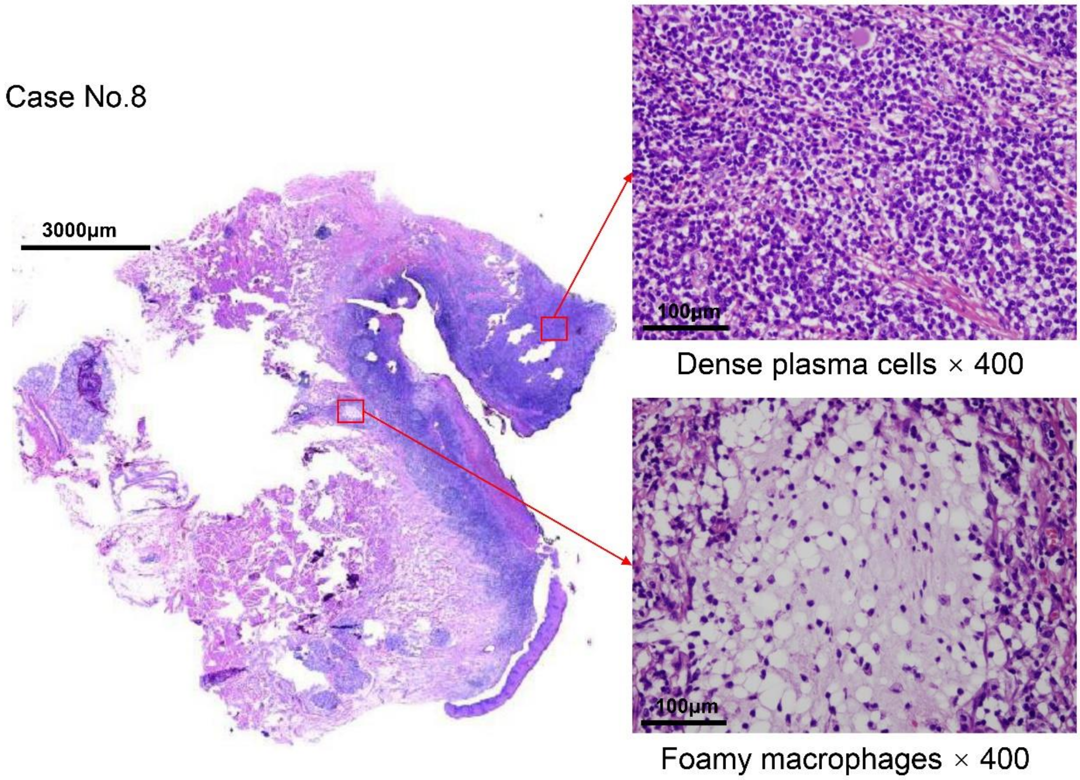

Supplementary Figure 9. Representative immune-related pathologic tumour bed HE pictures (Case No.8).

A representative H&E staining image of case No.8 shows: dense plasma cells and foamy macrophages. H&E, haematoxylin and eosin-stained.

Supplementary Note 1. Study protocol

Inductive Camrelizumab and Apatinib for Patients with Locally Advanced and Resectable Oral Squamous Cell Carcinoma

Version number/version date: Version 1.1/August 15, 2020

Trial sponsor: Ninth People's Hospital, Shanghai Jiao Tong University School of Medicine

Statistical analyst division: Ninth People's Hospital, Shanghai Jiao Tong University School of Medicine

Principal investigator: Lai-ping Zhong

Trial start time: March 1, 2020

Version/revision history

File	Version date	Explanation of the reason for the amendment and summary of the amendment
Version 1.0	Jan. 9, 2020	**The reason for the amendment:** CT and/or MRI after the completion of inductive therapy should be performed before surgery. The time window in the original version wasn't described exactly.<br><br>**The summary of the amendments: "**After the third cycle of inductive therapy (D14 ± 7 days of the third cycle), oral, maxillofacial and neck regions will be assessed by enhanced CT and/or MRI." has been revised to "After the third cycle of inductive therapy, oral, maxillofacial and neck regions will be assessed by enhanced CT and/or MRI before surgery." **Trial procedures - Treatment and observation period - Inductive therapy - Frequency of examination (page 7, page 16)**
Version 1.1	Aug. 15, 2020	

Signature page of the main investigator (group leader unit)

I will conscientiously perform my duties as an investigator in accordance with China's Good Clinical Practice (GCP) and personally participate in or directly guide this clinical trial. I have received the investigator's manual of the investigational drug in this clinical trial. I have read and understand the preclinical research status of the investigational drug and the research protocol for this clinical trial. I agree to perform relevant duties in accordance with Chinese laws, the Declaration of Helsinki, China's GCP and this research protocol. Unless measures must be taken to protect the safety, rights and interests of the subjects, I will only make changes to the protocol after notifying the sponsor and obtaining consent and will implement changes after approval by the Ethics Committee. I will be responsible for making clinical medical decisions, ensuring that subjects receive appropriate treatment in a timely manner when adverse events occur during the study and documenting and reporting these adverse events in accordance with relevant national regulations. I promise to document the data in a true, accurate, complete and timely manner in the study database. I will willingly accept supervision and inspections by personnel dispatched by the sponsor and inspections by the drug supervision and management department to ensure the quality of clinical trials. I promise to keep each subject's personal information and related matters confidential. Before the start of the study, I will provide the principal investigator's resume, which will be submitted to the ethics committee and to the drug regulatory department.

Research Institute: Ninth People's Hospital, Shanghai Jiao Tong University School of Medicine

Principal Investigator: Lai-ping Zhong (print) __________________ (signature)

Date of Signature: ________________________

Contact Number: ______________

Address: Floor 13, Building 1, No. 639, Zhizaoju Road, Shanghai

Zip Code: 200011

I. Summary

Title: Inductive Camrelizumab and Apatinib for Patients with Locally Advanced and Resectable Oral Squamous Cell Carcinoma

Trial stage: Phase I
Sponsor: Ninth People's Hospital, Shanghai Jiao Tong University School of Medicine
Responsible party: Ninth People's Hospital, Shanghai Jiao Tong University School of Medicine
Principal investigator: Lai-ping Zhong
Indication: Oral squamous cell carcinoma
Trial drugs: Camrelizumab (200 mg/vial) and Apatinib (250 mg/tablet)
Objective: To determine the safety and pathological efficacy of inductive therapy using a combination of anti-PD1 and anti-VEGFR inhibitors for patients with locally advanced resectable OSCC.
Subjects: Patients with locally advanced resectable oral squamous cell carcinoma
Number of subjects planned to be enrolled: 20 patients
Inclusion criteria: 1. Age: 18-75 years old

2. Gender: male and female
3. Eastern Cooperative Oncology Group (ECOG) performance status (PS): 0-2
4. Histopathological diagnosis of oral squamous cell carcinoma (including tongue, gums, cheek, floor of mouth, hard palate, and posterior molar region)
5. Primary tumour with a clinical stage of III/IVA (T1-2/N1-2/M0 or T3-4a/cN0-2/M0, AJCC 2018)
6. Blood routine: white blood cells (WBCs) >3,000/mm^3, hemoglobin >8 g/L, platelets >80,000/mm^3
7. Liver function: alanine amino transferase/aspartate amino transferase (ALAT/ASAT) <2.5 times the upper limit of normal and bilirubin <1.5 times the upper limit of normal
8. Renal function: Serum creatinine <1.5 times the upper limit of normal
9. Signed the informed consent form

Exclusion criteria: 1. Unresolved grade 2 [(Common Terminology Criteria for Adverse Events (CTCAE 5.0)] or higher toxic reactions caused by previous anticancer treatments

2. Obvious cardiovascular abnormalities [such as myocardial infarction, superior vena cava syndrome, grade 2 or higher heart disease diagnosed according to the New York Heart Association (NYHA) classification 3 months before enrollment]
3. Active severe clinical infection (> National Cancer Institute (NCI)-CTCAE version 5.0 grade 2 infection)
4. Uncontrollable hypertension (systolic blood pressure >150 mmHg and/or diastolic blood pressure >90 mmHg) or cardiovascular diseases with clinical significance (such as activity), such as cerebrovascular accidents (≤ 6 months before screening), myocardial infarction (≤6 months before screening), unstable angina pectoris, NYHA grade II or above congestive heart failure, or severe arrhythmia that cannot be controlled by drugs or has a potential impact on trial treatment.
5. Pregnant or lactating women
6. Participation in other clinical trials within 30 days before enrollment
7. Other situations that the investigator considers unsuitable with respect to participating in the trial

Trial design: single-arm, single-center, prospective clinical trial

Trial procedures: screening period, treatment and observation period, and follow-up period.

Screening period:

Eligible patients were enrolled and began receiving drug treatment.

Treatment and observation period:

Inductive therapy

Inductive therapy: the patients received three cycles of intravenous Camrelizumab (anti-PD1 inhibitor, 200 mg) on d1, d15 and d29; and oral Apatinib (anti-VEGFR inhibitor, 250 mg) daily, starting on d1 and ending on the 5th day before surgery.

Radical surgery was performed on the 42th-45th after initiation of inductive therapy. Post-operative radiotherapy was planned within 1.5 months after surgery, depending on the clinical and pathological stage.

During the inductive therapy, if rapid disease progresses or unacceptable toxic reaction occurs, inductive therapy will be terminated. After necessary treatment, surgical treatment will be performed as soon as possible.

During the treatment period, at the beginning and end of each treatment cycle, the subjects will be required to visit the hospital to report concomitant medications, evaluate adverse events, and complete the following laboratory

examinations and assessments of tumour treatment efficacy.

Surgical treatment stage

The patient will receive surgery on the 42th-45th after initiation of inductive therapy. The preoperative examination will include a physical examination, vital signs, complete blood count, routine urinalysis, blood biochemistry, blood electrolytes, surgical records, assessment of pathological efficacy, adverse reactions during surgical treatment, and concomitant medication for the treatment of adverse reactions.

Postoperative therapy (within 4-6 weeks after the end of surgical treatment)

Complete blood count, routine urine, blood biochemistry and blood electrolyte tests, radiotherapy regimen records, chemotherapy drug use records, concomitant medication records and reports of adverse events during radiotherapy/radiochemotherapy will be required. If necessary, tumour imaging evaluations will be performed.

Follow-up period:

The follow-up period will be two years. CT and/or MRI will be performed at least every six months, and the survival status of and local tumour recurrence in the patients will be recorded. The first follow-up will be within 30 days after the completion of treatment, and follow-up will be conducted every three months to record tumour information, survival information, ECOG score, concomitant medication/treatment, and adverse events. Enhanced CT and/or MRI for oral and maxillofacial and neck regions and chest CT will be performed every six months after the surgery for imaging assessments; additionally, if the risk of tumour recurrence or metastasis is revealed during follow-up, CT and/or MRI will be performed.

Primary endpoints:

- **Adverse events:** adverse events during the study period will be evaluated according to the NCI-CTCAE, version 5.0.
- **Pathological efficacy:** the percentage of viable tumour cells in resected tumour specimens after inductive therapy.

Secondary endpoints: two-year overall survival rate, local tumour recurrence rate.

II. Purpose of the trial

A single-arm clinical trial will be conducted to investigate the pathological response efficacy of inductive therapy using anti-PD1 plus anti-VEGFR for patients with locally advanced resectable oral squamous cell carcinoma. This trial will lay a good foundation

for the implementation of further randomized controlled trials.

III. Trial design

Single-center, single-arm, prospective clinical trial.

IV. Selection of subjects

1. Inclusion criteria: see previous content.

2. Exclusion criteria: see previous content.

3. Criteria for termination of the trial:

Termination of the trial refers to the early termination of the treatment specified in the protocol during the clinical trial. The main purpose is to protect the rights and interests of the subjects, to ensure the quality of the trial and to avoid unnecessary economic losses.

If one of the following conditions is met, the trial will be terminated:

- A serious safety problem during the trial
- A major error in the protocol during the trial, making it difficult to evaluate the endpoint indicators, or a well-designed protocol with significant deviations in implementation, making it difficult to evaluate the endpoint indicators if the trial continues
- Termination request by the investigator (e.g., funding reasons, management reasons, etc.)
- Termination request by the administrative department or the ethics committee
- Investigators believe that continuation of the trial may harm the interests of the subjects

4. Withdrawal from the trial:

Patients have the right to withdraw from the trial at any time for any reason. Investigators will contact the patients via telephone or follow-up visits or to fully understand the reasons for withdrawal through their relatives and document reasons on the case report form (CRF).

The investigators will also have the right to decide to withdraw a subject from the trial in the event of relapse of the pre-existing disease, the occurrence of serious adverse events that preclude continuation of the trial, violation of the treatment regimen, poor compliance by the subjects, taking other drugs during the trial that interfere with the efficacy evaluations, and management issues or other reasons. The withdrawal of too many subjects will lead to unreliable trial results; therefore, unnecessary withdrawal will be avoided. If a patient withdraws from the

study due to the occurrence of adverse events or abnormal laboratory test results, it will be recorded in the CRF. Patients who withdraw early will not be replaced by other subjects.

V. Experimental drugs and management

1. Trial drug 1: Camrelizumab

Manufacturer: Jiangsu Hengrui Pharmaceuticals Co., Ltd.

Dosage form: lyophilized powder

Specification: the specified dose of this drug is 200 mg, and it is packaged in 20 mL vials (batch number can be found in the drug test report).

Direction for use: intravenous injection

Expiration date: three years from the date of manufacture

Storage conditions: drug should be kept sealed, protected from light and placed in a 2-8 °C medical refrigerator

2. Trial drug 2: Apatinib mesylate tablets

Manufacturer: Jiangsu Hengrui Pharmaceuticals Co., Ltd.

Dosage form: tablet

Specification: 250 mg/tablet

Direction for use: take orally after meals (at the same time every day)

Expiration date: three years

Storage conditions: sealed, protected from light, and stored below 25 °C

3. Administration method

There will be three cycles of inductive therapy, 14 days each cycle. The dose and administration method are as follows: 200 mg of Camrelizumab will be administered by intravenous infusion on the first day of each cycle; 250 mg of Apatinib will be orally administered once per day from the first day of each cycle until the end of the cycle for the first two cycles; and then, drug administration will be stopped 5 days in advance in the third cycle.

4. Concomitant medication

The medication history of the subjects within the 14 days prior to screening and the concomitant treatment information during the entire trial period will be collected. All concomitant medications and treatment methods will be documented in detail in the CRF, and the reason for medication, medication dose, and medication time will be noted.

- During the entire trial, the subjects will not be allowed to use antitumour drugs other than the trial drugs.

- Researchers can take appropriate supportive treatment after assessing the relationship between adverse events and medication. The start and duration of supportive treatment, e.g., antiemetics, antidiarrheals, antipyretics, antihistamines, analgesics, antibiotics, and other blood products, will be documented in the CRF.
- Maintenance therapy will be provided for underlying conditions (such as hypertension, diabetes, etc.). Medications and reasons for administration will be fully documented in the concomitant medication section of the CRF.
- During the trial period, subjects will be prohibited from using modern Chinese medicine preparations and immunomodulators (including but not limited to interferon, interleukin-2, thymosin, etc.) approved by the National Medical Products Administration (NMPA) for the treatment of oral cancer.

During the trial period, subjects will not be allowed to receive any local treatment targeting oral cancer lesions, and other systemic antitumour therapies, such as chemotherapy, molecular targeted therapy, steroid therapy, immunotherapy, and traditional Chinese medicine treatment, will not be allowed.

Drugs to be used with caution during the trial:

- Drugs that interfere with cytochrome P450 enzymes in the liver:
  - Cytochrome P450 3A4 (CYP3A4) inducers: dexamethasone, phenytoin, carbamazepine, rifampicin, phenobarbital, rifapentin, etc.
  - CYP3A4 inhibitors: itraconazole, clarithromycin, voriconazole, telithromycin, saquinavir, etc.
  - Drugs metabolized by CYP3A4: benzodiazepines, dihydropyridine, calcium channel blockers (nisoldipine and lercanidipine)
  - Hydroxymethylglutaryl-CoA (HMG-CoA) reductase inhibitors: simvastatin and midazolam
  - Drugs metabolized by CYP2C9: warfarin, phenytoin and certain sulfonylureas, such as glibenclamide
  - Drugs that prolong the QT interval include antibiotics, antiarrhythmic drugs, antipsychotic drugs, antifungal drugs, antimalarial drugs, and antidepressants
  - Drugs allowed to be used in combination during the trial:
- Steroids

Local application of steroids, such as topical, eye, nasal, intra-articular,

inhalation, etc., will be allowed, as will corticosteroids for adrenal replacement therapy; corticosteroids for the treatment of adverse reactions; and steroids for the short-term prevention and treatment of allergic reactions (prevention of contrast agent allergies or treatment of other allergic reactions).

Other systemic treatments: during treatment, subjects will be provided the best supportive treatment. Hormone replacement therapy will be allowed. Bisphosphonates will be allowed for the treatment of bone metastases.

Palliative local treatment: palliative treatment will be allowed for local nontarget lesions that cause obvious symptoms, such as bone lesions with pain. Local radiotherapy or surgery can be considered, but the following conditions must be met:

- For subjects who require local treatment due to aggravation of symptoms during the trial, the investigator must determine whether the disease has progressed
- Subjects with disease progression must meet the criteria for continued treatment after progression
- Lesions treated with local therapy cannot be the target lesions

A discussion with the investigator is recommended before starting palliative local treatment. The content of palliative treatment will be documented in detail in the CRF and medical records, including the treatment date, location, treatment method and dose, and adverse reactions.

5. Drug distribution and management

All drugs in the trial institution will be managed by a special drug administrator to ensure that the drugs are only used for qualified subjects in the clinical trial. The distribution of trial drugs to non-enrolled individuals is prohibited. The clinical research associate (CRA) is responsible for monitoring the supply, use, and storage of drugs used in clinical trials and the disposal process of residual drugs.

The trial drugs will be stored in a locked medicine cabinet as required.

The investigators will be responsible for the inventory, verification and recording of the trial drugs. The investigators or designated personnel must maintain a record of the number of drugs during the entire trial process. Missing drugs will be recorded, i.e., the trial drugs will be kept and distributed by a designated person in the clinical trial institute, and the process of accepting, receiving and returning will be recorded in detail.

Once the study is completed, for all unused drugs that are not authorized by the sponsor for local destruction, the CRA will retrieve the drugs along with the corresponding use records.

VI. Study procedure

In this study, Camrelizumab and Apatinib will be used. Subjects who meet the protocol will be treated with the trial drugs. The entire trial will be divided into a screening period, treatment and observation period, and follow-up period.

Screening period:

- Medical history: medical history, prescreening treatment and other comorbidities, and medication history
- Physical examination: height, weight, blood pressure, and heart rate
- Tumour assessment: tumour assessment results 21 days before screening can be used for baseline screening. Tumour assessment can be performed by computed tomography (CT) and/or magnetic resonance imaging (MRI). It is required that the same subject use the same assessment method during the trial period.
- ECOG score
- Quality of life questionnaire
- Complete blood count
- Blood biochemical test
- Blood electrolyte test: potassium, sodium and chloride
- Routine stool and occult blood test
- Routine urinalysis: WBCs, urinary protein, and red blood cells
- Coagulation function
- Standard 12-lead electrocardiogram (ECG)
- Pregnancy test: suitable for women of childbearing age
- Hepatitis B, hepatitis C, human immunodeficiency virus (HIV), and toluidine red unheated serum test (TRUST) viral marker test

Eligible patients were enrolled and began receiving drug treatment.

Treatment and observation period:

Inductive therapy

Inductive therapy: there will be a total of three cycles, with 14 days in each cycle. The dose and administration methods will be as follows: 200 mg of Camrelizumab will be administered by intravenous infusion on the first day of each cycle; 250 mg of Apatinib will be administered orally once a day from the first day of each cycle until the end of the cycle for the first two cycles; and then, drug administration will be stopped on the 5th day before surgery.

During inductive therapy, if the rapid disease progresses or unacceptable

toxic reaction occurs, inductive therapy will be terminated. After the corresponding treatment, surgical treatment will be performed as soon as possible.

At the beginning and end of each treatment cycle, the subjects will be required to visit the hospital to report concomitant medications, evaluate adverse events, and complete the following laboratory examinations and assessments of tumour treatment efficacy.

Examination content:

- Complete blood count
- ECOG score
- Routine urinalysis
- Blood biochemical test
- Blood electrolytes: potassium, sodium and chloride
- Coagulation function: prothrombin time (PT), activated partial thromboplastin time (APTT), and international normalized ratio (INR)
- CT or MRI assessment of tumour remission
- Other indicators that investigators think need to be examined
- Physical examination: height, weight, blood pressure, and heart rate

Frequency of examination: the following assessments will be completed within ±5 days of D1 in each treatment cycle: physical examination results, ECOG score, vital signs, complete blood count, urine test, blood biochemistry, blood electrolytes, and coagulation test. Assessments will be reviewed before initiating any treatment. After the third cycle of inductive therapy, oral, maxillofacial and neck regions will be assessed by enhanced CT and/or MRI before surgery.

Surgical treatment stage

Radical surgery was performed on the 42th-45th after initiation of inductive therapy. The preoperative examination will include a physical examination, vital signs, complete blood count, routine urinalysis, blood biochemistry, blood electrolytes, surgical records, assessment of pathological efficacy, adverse reactions during surgical treatment, and concomitant medication for the treatment of adverse reactions.

Surgical resection generally involves en bloc resection of the primary tumour with 1cm - 1.5cm tumour-free margins, combined with neck dissection based on cN stage. Reconstruction with microvascular free flaps will be performed if the defect is obvious. Clavien-Dindo grading tool will be used for

surgical complications assessment (Appendix - Clavien-Dindo Grading).

Postoperative therapy (within 4-6 weeks after the end of surgical treatment)

Complete blood count, routine urine, blood biochemistry and blood electrolyte tests, radiotherapy regimen records, chemotherapy drug use records, concomitant medication records and reports of adverse events during radiotherapy/radiochemotherapy will be required. If necessary, tumour imaging evaluations will be performed.

Follow-up period: the follow-up period will be two years. CT and/or MRI will be performed at least every six months, and the survival status of and local tumour recurrence in the patients will be recorded. The first follow-up will be within 30 days after the completion of treatment, and follow-up will be conducted every three months to record tumour information, survival information, ECOG score, concomitant medication/treatment, and adverse events. Enhanced CT and/or MRI for oral and maxillofacial and neck regions and chest CT will be performed every six months after the surgery for imaging assessments; additionally, if the risk of tumour recurrence or metastasis is revealed during follow-up, CT and/or MRI will be performed.

Study flow chart

	Screening period	Inductive therapy	Surgical treatment	Postoperative treatment	Follow-up period
Examination item	-3 w~0 d	0 w-6 w			If necessary
Informed consent	**X**	-			
Medical history	**X**	-			
Pregnancy test [1]	**X**	-			
Vital signs	**X**	Once/cycle	**X**	**X**	
Physical examination	**X**	Once/cycle	**X**	**X**	
Complete blood count	**X**	Once/cycle	**X**	**X**	
Routine urinalysis	**X**	Once/cycle	**X**	**X**	
Routine stool and occult blood test [2]	**X**	If necessary	**X**	**X**	
Blood biochemistry [3]	**X**	Once/cycle	**X**	**X**	
Coagulation function [4]	**X**	Once/cycle	**X**	**X**	
Hepatitis B, hepatitis C, HIV, and TRUST viral marker tests	**X**	-			
Tumour evaluation [5]	**X**	Before surgery	**X**	**X**	
12-lead ECG	**X**	If necessary	**X**	If necessary	
B-mode cardiac ultrasound [6]	**X**	If necessary	**X**	If necessary	
Enhanced CT scan or MRI examination of oral and maxillofacial and neck regions	**X**	Before surgery	**X**	**X**	
Assign subject	**X**	-			

identification number					
Dosing		200 mg of Camrelizumab will be administered via intravenous infusion once every two weeks, for a total of three times. One 250 mg capsule of Apatinib will be given daily until five days in advance of the surgery			
Concomitant medication	X	X	X	X	
Record adverse events	X	X	X	X	

Note: 1. Pregnancy test: only applicable to women of childbearing age. 2. Routine stool and occult blood tests: only performed during the screening period and during the preoperative examination. If examinations are required during treatment, physicians will request laboratory tests on a case-by-case basis. 3. Blood biochemistry: ALAT, ASAT, total bilirubin (TBIL), direct bilirubin (DBIL), indirect bilirubin (IBIL), blood urea nitrogen (BUN), creatinine (Cr), creatinine clearance (Ccr), gamma-glutamyl transferase (GGT), albumin (ALB), potassium, sodium, and chloride. 4. Coagulation function: PT, APTT, INR. 5. Tumour assessment will be performed using CT and/or MRI, but the same subject must receive the same examination method. 6. B-mode cardiac ultrasound to assess cardiac function.

VII. Trial endpoint:

Primary endpoints:

- **Adverse events:** adverse events during the study period will be evaluated according to the NCI-CTCAE, version 5.0.
- **Pathological efficacy:** the percentage of viable tumour cells in resected tumour specimens after inductive therapy.

Secondary endpoints: two-year overall survival rate, local tumour recurrence rate

VIII. Adverse events and serious adverse events

1. Adverse events

Adverse events (AEs) refer to any adverse signs, symptoms or medical conditions that occur or are aggravated after the use of trial drugs, even if the event is considered unrelated to the trial drugs. Medical conditions/diseases that exist before the start of the trial will be recorded as an AE only if they are aggravated after the use of trial drugs. Abnormal laboratory results will be considered AEs only when they cause clinical symptoms or signs, are considered to have significant clinical significance, or require intervention.

In this trial, AEs will be recorded right after signing the informed consent form. Any serious AEs that occur 30 days after the termination of the treatment will be reported only when the investigator suspects that such AEs are related to the intervention of this trial.

During their stay in the research center, subjects will be asked regularly about the occurrence of AEs (using neutral questions, such as “how do you feel?”).

The AEs spontaneously reported by the subjects during the trial period and/or revealed by responses to questions and the AEs found in the physical examination, laboratory examination or other evaluations will be recorded in an CRF adverse event form, and severity (mild, moderate, and severe), onset time, end time, duration, treatment measures (including measures for trial drugs), outcome, relationship with trial drugs, and whether it is a serious adverse event (SAE), etc. will be specified. AEs that occur after signing the informed consent but before the start of the study treatment will be recorded on the previous history/existing disease history page of the CRF. All AEs will be followed up until they are properly resolved or the condition is stable.

Adverse drug reactions (ADRs) refer to AEs associated with any dose of the trial drugs. All AEs that the investigators judge to have a reasonable causal relationship with the trial drugs will be considered ADRs.

1.1 Severity of AEs

The severity of AEs will be assessed using the CTCAE, version 5.0:

Grade 1: mild; asymptomatic or mild, only clinical or diagnostic observable events; no treatment is needed

Grade 2: moderate; requires minor, local or noninvasive treatment; age-appropriate limitation in instrumental activities of daily living

Grade 3: severe or medically significant but not immediately life-threatening; leading to hospitalization or prolonged hospitalization; disability; limitation of personal activities of daily living

Grade 4: life-threatening; emergency treatment is required

Grade 5: AE-related death

Note: instrumental activities of daily living refer to cooking, buying clothes, using the phone, managing finances, etc. Personal activities of daily living include bathing, dressing and undressing, eating, washing, taking medication, etc., without being bedridden.

1.2. Causality assessment

The following terms will be used to describe the causal relationship between AEs and trial drugs:

Definitely related: a clinical event includes laboratory abnormalities, the occurrence of which has a reasonable sequential relationship with the application of trial drugs and cannot be explained by concomitant diseases or other drugs. The drug withdrawal reaction is clinically reasonable. This event must have a positive correlation with the trial drugs in terms of pharmacological or clinical manifestations and reappear when the drug is administered again.

Most likely related: a clinical event includes laboratory abnormalities, the occurrence of which has a reasonable sequential relationship with the application of trial drugs and is unlikely to be caused by concomitant diseases or other drugs. The drug withdrawal reaction is clinically reasonable. This event does not necessarily occur when the drug is administered again.

May be related: a certain clinical event includes laboratory abnormalities, the occurrence of which has a reasonable sequential relationship with the application of trial drugs but can also be explained by concomitant diseases or other drugs. Drug withdrawal information can be missing or unclear.

May be unrelated: a certain clinical event includes laboratory abnormalities, the occurrence of which has a sequential relationship with the application of trial drugs but may not have a causal relationship with the trial drugs. Other drugs or diseases can provide a reasonable explanation for the causal relationship.

Unevaluable: AEs that do not meet any of the above criteria.

1.3. The outcome of AEs

The following terms are used to describe the outcome of AEs:

- Recovery
- Recovering
- Recovery with sequelae
- Not recovered
- Death
- Unknown

1.4. Follow-up of AEs

If an AE occurs at the end of the trial or continues, the investigator will continue to follow-up until the event disappears or the condition is stable.

2. SAEs

SAEs are defined as events that lead to at least one of the following outcomes:

- Death
- Life-threatening
- Need to be hospitalized or extend the length of hospitalization
- Persistent or significant disability/inability to work
- Congenital anomalies/birth defects

Other important medical events: although not immediately life-threatening or a direct cause of death or hospitalization, they may endanger the health of subjects or may require medical intervention to prevent the occurrence of any important AE/ADR.

Researchers will immediately take appropriate treatment measures for all subjects

with SAEs to ensure the safety of the subjects and document the treatment measures and the progress of the events. If the occurrence of an SAE is determined by the investigator to be related to the trial drug, the sponsor will provide certain financial compensation in accordance with the relevant national laws and regulations.

The investigators will report any SAE that occurs after the subjects sign the informed consent form until within 30 days after the termination of the trial, regardless of whether the SAEs are related to the trial drug, to the sponsor within 24 hours after being informed of the occurrence and report SAEs to the Ethics Committee and National Medical Products Administration (NMPA) by fax as required by GCP. Any SAE that occurs 30 days after the termination of the trial will be reported only when the investigator suspects that such SAE is related to the trial drugs.

Investigators will collect all the information on SAEs and document them in an SAE report form. Investigators will assess the correlation between SAEs and trial drugs, complete and sign the SAE report form, and fax the completed and signed report form to the sponsor within 24 hours. The original SAE report form and fax confirmation will be retained at the clinical trial center.

Within 24 hours of receiving follow-up information, investigator must report recurrence, complications, or progression of the original SAE as a follow-up event of the original event. SAEs that occur at different time intervals and are considered to be completely unrelated to previously reported SAEs will be reported as new SAEs. The follow-up report will explain whether the AEs are resolved or continue, whether treatment is needed and how it is performed, whether unblinding is necessary, and whether the subject should continue the trial. The sponsor can raise questions to clarify the details of SAEs.

IX. Statistical analysis

Overall survival will be calculated from the date of enrollment until death using the Kaplan-Meier method. The confidence interval (CI) of the survival rate and local recurrence rate will be calculated using the Clopper-Pearson method. Linear regression analysis will be performed to evaluate the concordance between the RVT value and the changes in the radiographic diameters of primary tumours. The P values of quantified fluorescence differences will be calculated between two groups using the Student's t-test. The significance level for two-sided P values is set at 0.05 in statistical analyses. Statistical analysis will be performed in IBM SPSS Statistics and GraphPad

Prism software.

X. Clinical trial Management

1. Approval by the ethics committee

The design, implementation and report of this clinical trial followed GCP, the current Helsinki Declaration, the relevant regulations of the NMPA and the opinions of the ethics committee.

Before the start of the trial, the investigator will obtain written approval of the ethics committee for the trial protocol, informed consent, subject recruitment procedures and other written information that will be provided to the subjects. During the trial period, if there are any new amendments to the trial protocol and informed consent form, written approval from the ethics review committee should be obtained again before implementation.

The composition and operation of the ethics committee will adhere to national regulations.

1. Informed consent

The investigators will be responsible for explaining to each subject the clinical trial background, the characteristics of the trial drug, the trial protocol, other treatment measures for related diseases, and the benefits and risks of participating in the trial, and a written informed consent form signed by each subject or his/her legal representative will be obtained before each subject participates in the trial (before the screening examination).

The informed consent text will include the following content: the purpose of the trial, the name and the characteristics of the trial drug, the ADRs, the dose, the number of doses, blood sample collection, the trial procedures, the obligations of the subjects, the compensation for the subjects to participate in the trial, and risks and inconveniences; treatment and appropriate compensation to the subjects in the event of trial-related harm; access to the trial data; and confidentiality of subject information.

The informed consent text will be written in a language that the subjects can read. The informed consent form will be approved by the ethics committee in writing. The informed consent form will be signed and dated by each subject or his/her legal representative and the investigators participating in the informed consent process. The investigator and the subject will each receive a copy of the informed consent form. If important new information related to the trial drug is found, the informed consent form will be modified in writing and sent to the ethics committee for approval, and then consent will be obtained again from each subject, using the aforementioned procedure,

before the subject resumes participation in the trial.

3. Data management

3.1. Database development

An electronic data acquisition system will be used for data entry and data management in this clinical trial. A database administrator will develop a database based on the protocol and CRF samples and prepare a quality control program for data entry in accordance with requirements.

An electronic data capture (EDC) system will be officially launched after testing by the database administrator, data administrator, investigator, clinical research coordinator (CRC), CRA, and medical personnel.

3.2 Data entry

Before the start of the trial, the data administrator will provide training on the use of the EDC system to the investigators in the research center or the CRC. After the start of the trial, the investigators or the CRC will enter the data once in real time.

3.3 Data approval

After the investigators or the CRC complete data entry, the CRA will conduct online raw data approval. The CRA, medical staff, and data administrator will verify the data and send data queries online. After the queries are sent, the investigators will verify the raw data and resolve questions online.

3.4 Medical coding

All AEs reported in the clinical trial will be medically coded using the "Medical Dictionary for Regulatory Activities by International Council for Harmonisation of Technical Requirements for Pharmaceuticals for Human Use (ICH)". Medical coding will be completed before the database is locked.

3.5 Data management report

When all the data are cleaned up and the database quality control rate meets requirements, the data administrator will prepare a data management report based on the protocol and actual operation of the project.

3.6. Database locking

The locked data file will not be able to be changed unilaterally. Problems discovered after the database is locked will be corrected in the statistical analysis feedback query form after confirmation by the principal investigator, statisticians, data administrators and sponsors and written signature for the record.

4. Trial monitoring

The sponsor will arrange for the CRA to visit the research institute regularly to conduct clinical monitoring. The investigators will actively cooperate with the CRA and will allow the CRA to have direct access to all the documents related to the trial, such

as CRFs, complete original documents and investigator documents. In addition, investigators will answer questions from CRA personnel and correct data in CRFs.

The following specific content will be included in CRA monitoring:

- Confirm that the trial center is appropriately equipped before the trial starts, including personnel allocation and training, various examinations related to the trial, a well-equipped laboratory, and good working conditions, that the number of subjects are sufficient, and that participating investigation personnel are familiar with the requirements of the trial protocol;
- Monitor the research institute and investigators before, during and after the trial, confirm that informed consent has been obtained from all subjects before the trial, understand the enrollment rate of subjects and the progress status of the trial, and supervise the implementation of the trial in strict accordance with the trial protocol and GCP;
- Confirm that all data records are correct and complete, all CRFs are filled in correctly and consistent with the original data, and all errors or omissions have been corrected or noted and signed and dated by the investigator;
- Confirm that AEs are documented and that SAEs are reported and documented within the specified time;
- Verify that the trial drugs are supplied, stored, distributed, and retrieved in accordance with relevant regulations and that all information are recorded accordingly; and
- Assist the investigators with the necessary notification and application matters and report the trial data and results to the sponsor.

5. Audit and inspection

In addition to regular monitoring procedures, the quality assurance department of the sponsor may also check whether the trial is conducted in accordance with GCP and the sponsor’s standard operating procedure (SOP) at any time. During the trial or after the trial, the relevant state departments may also conduct inspections.

6. Revision of the protocol

The implementation of this trial will completely follow the trial protocol, GCP, the current Helsinki Declaration, the relevant regulations of the NMPA and the opinions of the ethics committee. During the implementation of the trial, any changes or additions to the protocol must be in the form of written protocol revision. The revised protocol can only be implemented after approval by the ethics committee.

7. Termination of the trial

The sponsor has the right to terminate this trial at any time for any reason. If it is

necessary to terminate the trial, the investigators will be informed of the steps to be followed to ensure that the interests of the subjects are fully protected. The investigators are responsible for reporting the early termination of the trial to the ethics committee.

8. Confidentiality

The information provided by the sponsor to the investigators is nonpublic, must be kept confidential and cannot be released to any organization or institution that is not directly involved in the trial.

Investigators must ensure the anonymity of subjects. Regarding patients protection, this clinical trial will follow the relevant GCP regulations. The medical data of all subjects and their lifestyles will be processed by computer and will only be transferred to the sponsor or the relevant departments of the NMPA under the condition of ensuring a high level of confidentiality.

XI. Quality control and quality assurance

The clinical trial institute is recognized by the NMPA with clinical trial conditions of a drug clinical trial institution.

Investigators will be trained in clinical trials, obtain appropriate qualifications and work under the guidance of senior professionals.

Before the trial, the clinical ward will meet the standardized requirements to ensure that the rescue equipment is fully equipped.

The subjects will be given drugs by professional nursing staff and understand the use of the drugs in detail to ensure participant compliance.

The trial protocol will be strictly followed, and the CRFs will be filled in truthfully.

The CRA should follow SOPs, supervise the progress of clinical trials, and confirm that all data records and reports are correct and complete and that all CRFs are filled in correctly and consistent with the original data to ensure that the trial is carried out in accordance with the clinical trial protocol.

Once an SAE occurs, the sponsor will temporarily stop the trial if necessary.

Each institute participating in the trial will be audited by the sponsor and the drug regulatory authority. Importantly, investigators and their related personnel will provide time for monitoring and auditing.

XII. Research progress

The enrollment period of this trial is expected to be 10 months, and the study period will be 34 months.

Planned date of enrollment of the first patient/start of the trial: March 1, 2020

Planned date of enrollment of the last patient: December 31, 2020

Planned date of the last patient released from the trial/end of trial: June 30, 2023

Planned database locking date: June 30, 2023

Estimated report date: September 30, 2023

XIII. Compliance with the trial protocol

The investigators promise to do their best to avoid protocol violations. If the investigator believes that a certain protocol deviation can improve the implementation of the trial, then protocol revisions will be considered, but revisions can only be implemented after approval by the Medical Ethics Committee. All major protocol violations will be documented and reported in the clinical trial report.

Appendix - Clavien-Dindo Grading

Grade 1: Any deviation from the normal postoperative course without the need for pharmacologic treatment or surgical, endoscopic, and radiologic interventions. Allowed therapeutic regimens are drugs as antiemetics,antipyretics, analgetics, and diuretics,and electrolytes and physiotherapy.This grade also includes wound infections opened at the bedside.

Grade 2: Requiring pharmacologic treatment with drugs other than such allowed for grade I complications.Blood transfusions and total parenteral nutrition are also included.

Grade 3: Requiring surgical, endoscopic, or radiologic intervention

3a: Intervention not under general anesthesia

3b: Intervention under general anesthesia

Grade 4: Life-threatening complication (including CNS complications) requiring IC/ICU management<a>

4a: Single organ dysfunction (including dialysis)

4b: Multiple organ dysfunction

Grade 5: Death as a result of complications

Abbreviations: CNS, central nervous system; IC, intermediate care; ICU,intensive care unit.

<a>Brain hemorrhage, ischemic stroke, or subarachnoidal bleeding but excluding transient ischemic attacks.
