# Supplementary material for: A pilot study of neoadjuvant combination of anti-PD-1 camrelizumab and VEGFR2 inhibitor apatinib for locally advanced resectable oral squamous cell carcinoma
Source: Nat Commun. 2022 Sep 14;13:5378. doi: 10.1038/s41467-022-33080-8 (PMC9472189; doi:10.1038/s41467-022-33080-8)
Supplement: Supplementary file 2 — Reporting Summary [file 41467_2022_33080_MOESM2_ESM.pdf]

nature portfolio

Corresponding author(s): Laiping Zhong

Last updated by author(s): Jul 24, 2022

Reporting Summary

Nature Portfolio wishes to improve the reproducibility of the work that we publish. This form provides structure for consistency and transparency in reporting. For further information on Nature Portfolio policies, see our Editorial Policies and the Editorial Policy Checklist.

Statistics

For all statistical analyses, confirm that the following items are present in the figure legend, table legend, main text, or Methods section.

n/a	Confirmed	
☐	☒	The exact sample size (*n*) for each experimental group/condition, given as a discrete number and unit of measurement
☐	☒	A statement on whether measurements were taken from distinct samples or whether the same sample was measured repeatedly
☐	☒	The statistical test(s) used AND whether they are one- or two-sided<br>*Only common tests should be described solely by name; describe more complex techniques in the Methods section.*
☒	☐	A description of all covariates tested
☐	☒	A description of any assumptions or corrections, such as tests of normality and adjustment for multiple comparisons
☐	☒	A full description of the statistical parameters including central tendency (e.g. means) or other basic estimates (e.g. regression coefficient) AND variation (e.g. standard deviation) or associated estimates of uncertainty (e.g. confidence intervals)
☐	☒	For null hypothesis testing, the test statistic (e.g. *F*, *t*, *r*) with confidence intervals, effect sizes, degrees of freedom and *P* value noted<br>*Give P values as exact values whenever suitable.*
☒	☐	For Bayesian analysis, information on the choice of priors and Markov chain Monte Carlo settings
☒	☐	For hierarchical and complex designs, identification of the appropriate level for tests and full reporting of outcomes
☐	☒	Estimates of effect sizes (e.g. Cohen's *d*, Pearson's *r*), indicating how they were calculated

Our web collection on statistics for biologists contains articles on many of the points above.

Software and code

Policy information about availability of computer code

Data collection	Data collection was performed in Microsoft Office Excel 2019.
Data analysis	Data analysis was performed in IBM SPSS Statistics (version 23), GraphPad Prism (version 9.1.2), and SAS (version 9.4) softwares.

For manuscripts utilizing custom algorithms or software that are central to the research but not yet described in published literature, software must be made available to editors and reviewers. We strongly encourage code deposition in a community repository (e.g. GitHub). See the Nature Portfolio guidelines for submitting code & software for further information.

Data

Policy information about availability of data

All manuscripts must include a data availability statement. This statement should provide the following information, where applicable:

- Accession codes, unique identifiers, or web links for publicly available datasets
- A description of any restrictions on data availability
- For clinical datasets or third party data, please ensure that the statement adheres to our policy

The trial protocol is available as Additional file 1 in the Supplementary Information file. Most de-identified clinical data of individual patients and multiplex immunofluorescence data are available in the manuscript or additional files. Other data can be obtained upon scientifically sound request from the corresponding author at zhonglp@hotmail.com. DNA data have been deposited in the Genome Sequence Archive for Human (https://ngdc.cncb.ac.cn/gsa-human/) under accession codes for HRA002175. Access to DNA data can be obtained upon scientifically sound request from the corresponding author at zhonglp@hotmail.com, who will contact the Clinical Research Unit, Ninth People's Hospital, College of Stomatology, Shanghai Jiao Tong University School of Medicine. Access will be released after the approval from the Clinical Research Unit.

Field-specific reporting

Please select the one below that is the best fit for your research. If you are not sure, read the appropriate sections before making your selection.

- [x] Life sciences
- [ ] Behavioural & social sciences
- [ ] Ecological, evolutionary & environmental sciences

For a reference copy of the document with all sections, see nature.com/documents/nr-reporting-summary-flat.pdf

Life sciences study design

All studies must disclose on these points even when the disclosure is negative.

Sample size	A sample size of 20 evaluable patients was required to achieve 90% power to detect an increase in the MPR rate from 7% (anti-PD-1 monotherapy, based on data from pembrolizumab and nivolumab monotherapy) to 30%, with a one-sided exact test with a significance level (alpha) of 0.050.
Data exclusions	No data were excluded from the analysis.
Replication	Due to limited sample of tumor biopsy, we only performed sequence/Immunohistochemistry/immunofluorescence once.
Randomization	It's a single-arm trial without randomization.
Blinding	It's a single-arm trial without blinding.

Reporting for specific materials, systems and methods

We require information from authors about some types of materials, experimental systems and methods used in many studies. Here, indicate whether each material, system or method listed is relevant to your study. If you are not sure if a list item applies to your research, read the appropriate section before selecting a response.

Materials & experimental systems

n/a	Involved in the study	
[ ]	[x]	Antibodies
[x]	[ ]	Eukaryotic cell lines
[x]	[ ]	Palaeontology and archaeology
[x]	[ ]	Animals and other organisms
[ ]	[x]	Human research participants
[ ]	[x]	Clinical data
[x]	[ ]	Dual use research of concern

Methods

n/a	Involved in the study	
[x]	[ ]	ChIP-seq
[x]	[ ]	Flow cytometry
[x]	[ ]	MRI-based neuroimaging

Antibodies

Antibodies used	For TILs evaluation: CD163 (Abcam, ab182422, 1:500), CD68 (Abcam, ab213363, 1:1000), PD-1 (CST, D4W2J, 86163S, 1:200), CD3 (Dako, A0452), CD4 (Abcam, ab133616, 1:100), CD8 (Abcam, ab178089, 1:100), CD56 (Abcam, ab75813, 1:100), CD20 (Dako, L26, IR604), FOXP3 (Abcam, ab20034, 1:100) and Pan-CK (Abcam, ab7753, 1:100). For vascular normalization evaluation: Anti-CD31 #ab178981 (dilution 1:1000, Abcam, USA), Alexa Fluor594 donkey anti-rabbit lgG(H+L) #A21207 (dilution 1:400, Life Technologies, USA), Anti-alpha smooth muscle Actin [1A4] #ab7817 (dilution 1:10000, Abcam, USA), Alexa Fluor488 donkey anti-mouset lgG(H+L) # A21202 (dilution 1:400, Life Technologies, USA), DAPI (dilution 1:500, #C0060, Solarbio, CHN). Among them, CD3 (Dako, A0452) and CD20 (Dako, L26, IR604) was used without any dilution.
Validation	All antibodies used in the study have been validated for the species and application.

Human research participants

Policy information about studies involving human research participants

Population characteristics	The characteristics of the 21 enrolled patients are listed in Table 1 of the manuscript.
Recruitment	This single-center, open-label trial was performed at the Ninth People's Hospital, Shanghai Jiao Tong University School of Medicine in Shanghai, China. Eligible patients were aged 18 to 75 years; had histopathological confirmed locally advanced OSCC with a clinical stage of III or Iva (American Joint Committee on Cancer, 8th Edition); and had an Eastern Cooperative Oncology Group performance status (ECOG PS) of 0 to 2. The full eligibility criteria are provided in the trial protocol (Supplementary Note 1). Each patient provided signed informed consent before participating in this trial.
Ethics oversight	The trial followed the ethical guidelines of the Declaration of Helsinki and was approved by the Institutional Ethics Committee, Ninth People's Hospital, Shanghai Jiao Tong University School of Medicine. Each patient provided signed informed consent before participating in this trial. An authorization of the release of the data was obtained from the Clinical Research Broad of Ninth People's Hospital.

Note that full information on the approval of the study protocol must also be provided in the manuscript.

Clinical data

Policy information about clinical studies

All manuscripts should comply with the ICMJEguidelines for publication of clinical research and a completedCONSORT checklist must be included with all submissions.

Clinical trial registration	The clinical trial registration number is NCT04393506.
Study protocol	The full eligibility criteria are provided in the trial protocol (Supplementary Note 1 in the Supplementary Information file).
Data collection	This single-center, open-label trial was performed at the Ninth People's Hospital, Shanghai Jiao Tong University School of Medicine in Shanghai, China. From April to December 2020, 21 patients were enrolled. Eligible patients were aged 18 to 75 years; had histopathological confirmed locally advanced OSCC with a clinical stage of III or IVA (American Joint Committee on Cancer, 8th Edition); and had an Eastern Cooperative Oncology Group performance status (ECOG PS) of 0 to 2. The authorization of the collection and release of the data from the Clinical Research Broad of our institution were obtained.
Outcomes	The primary endpoints were safety and major pathological response (MPR) rate (MPR, defined as the presence of 10% or fewer RVT cells). The 2-year survival rate (defined as the proportion of patients alive at the 2-year follow-up) and local recurrence rate (defined as the proportion of patients with local recurrence) were the secondary endpoints. Adverse events (AEs) were assessed according to the Common Terminology Criteria for Adverse Events (CTCAE, version 5.0). Neoadjuvant therapy-related AEs were managed mainly according to the American Society of Clinical Oncology Clinical Practice Guidelines. Surgery-related AEs were assessed with the Clavien-Dindo Classification of Surgical Complications.
